# Addressing Tobacco in Managed Care: Results of the 2003 Survey

**Published:** 2006-06-15

**Authors:** Carol McPhillips-Tangum, Bob Rehm, Rita Carreon, Caroline M Erceg, Carmella Bocchino

**Affiliations:** Experion Healthcare Group; America’s Health Insurance Plans, Washington, DC; America’s Health Insurance Plans, Washington, DC; America’s Health Insurance Plans, Washington, DC; America’s Health Insurance Plans, Washington, DC

## Abstract

**Introduction:**

Although tobacco control activity in the United States during the past several years has increased dramatically, tobacco use continues to have devastating consequences among all age cohorts.

**Methods:**

In November 2003, a survey of tobacco control practices and policies in health insurance plans was conducted by America's Health Insurance Plans' national technical assistance office. The survey was the fourth and final survey conducted as part of the Addressing Tobacco in Managed Care program. Of the 215 plans in the sample, 160 (74%) completed the survey. Collectively, these plans represent more than 60 million members of health maintenance organizations.

**Results:**

From 1997 to 2003, health insurance plans have demonstrated increasing use of evidence-based programs and clinical guidelines to address tobacco use. The number of plans providing full coverage for any type of pharmacotherapy for tobacco cessation has more than tripled since 1997. Plans have also shown substantial improvement in their ability to identify all or some of their members who smoke. Similarly, a greater percentage of plans are using strategies to address smoking cessation during treatment for other chronic diseases and after acute events such as a myocardial infarction.

**Conclusion:**

Despite improvements, important opportunities remain for health insurance plans and other stakeholders to expand their tobacco control activities and transfer the lessons learned to other health problems.

## Introduction

The scope and pace of tobacco control activity in the United States during the past several years has increased dramatically. Beginning with the 1998 multistate tobacco settlement, there has been heightened attention on tobacco use and an emergence of effective tobacco control strategies. Still, tobacco use continues to have devastating consequences among all age cohorts in the United States. New estimates from the Centers for Disease Control and Prevention (CDC) indicate that from 1997 to 2001, approximately 438,000 people in the United States died prematurely each year as a result of smoking or exposure to secondhand smoke (1). The most recent report from the U.S. surgeon general confirms that smoking harms almost every organ in the body, causing numerous diseases and reducing overall quality of life and life expectancy (2). Despite these grim facts, approximately 23% of American adults continue to smoke cigarettes (3).

In addition to the health consequences of tobacco use, it has enormous financial consequences. In 1998, smoking-attributable health care expenditures were estimated at $75.5 billion (4). More recent data from 1997 to 2001 indicate that smoking costs the United States approximately $92 billion annually in lost productivity (1). Together, the health and financial consequences of tobacco dependence continue to make treatment and prevention of tobacco use a priority among multiple stakeholders, including health plans, insurers, providers, employers, and policymakers.

In 1997, the Robert Wood Johnson Foundation (RWJF) established a collaborative program, Addressing Tobacco in Managed Care (ATMC). The program was based on the understanding that health insurance plans' comprehensive benefits, sophisticated information systems, defined populations, and partnerships with health care providers are well suited to implement, evaluate, and sustain tobacco control interventions. The initiative consisted of a national program office (NPO) located at the University of Wisconsin and the University of Illinois at Chicago and a national technical assistance office (NTAO) managed by America's Health Insurance Plans (AHIP). The mission of the NTAO was to advance the integration of tobacco cessation and prevention strategies into routine health care by increasing the number and quality of tobacco control initiatives within health insurance plans.

As part of the program, the NTAO provided resources to health insurers who were striving to develop tobacco control programs, conducted a benchmarking awards program to highlight exemplary health plan tobacco control initiatives, promoted best practices and partnerships through national conferences, and oversaw the development of a business case model for smoking cessation. The NTAO also conducted four surveys of health plans to assess practices and policies related to tobacco control. ATMC concluded its work in fall 2005.

The ATMC baseline survey was conducted in 1997, followed by similar surveys in 2000 and 2002. The results of the surveys were published in 1998, 2002, and 2004 (5-7). The fourth ATMC survey was conducted in 2003. This paper presents the results of the 2003 survey, highlights changes from 1997 to 2003, and explores findings and trends in light of tobacco control activities in the United States during the same period.

## Methods

A 32-item survey instrument was developed and pilot tested in fall 2003. The instrument was designed to assess new trends, barriers, and opportunities related to addressing tobacco control in health insurance plans, identify new models or frameworks of care, and assess changes in health-plan–based tobacco control activities between 1997 and 2003. The sample for the survey was drawn from the 687 health insurance plans listed in AHIP's national database of member and nonmember plans. The database was stratified based on plan enrollment size, and a random sample of 247 plans was selected. The sample size enables the detection of a 5% difference between proportions at α = .05 and β = .80.

The ATMC survey was conducted in winter 2003. As in 1997, 2000, and 2002, the 2003 ATMC survey was conducted through mail, e-mail, and fax, with telephone follow-up with nonrespondents at 2 weeks, 4 weeks, and 6 weeks after initial contact. The sample included large national plans that have local plans represented in multiple states. As in previous years, the corporate office of each national plan was asked to review the questionnaire and determine whether they would respond on behalf of their local plans or ask their local plans to complete the questionnaires individually. Three of four national plans opted to respond on behalf of their local plans, and their responses reflect 49% (78/160) of the responses. (The three national plans did not necessarily provide identical responses to all the survey questions for all their local plans.)

The 2003 survey questionnaire was longer but similar to the one used in 2002. Of the 32 items in the 2003 questionnaire, 17 were the same as in previous years; seven were added to collect more detailed data on areas of interest (e.g., pharmaceutical coverage, attributes of cessation interventions, strategies for notifying members about cessation benefits, member incentives); two were added to collect data on plans' use of return-on-investment (ROI) analysis and interest in ROI analysis tools; two were added to gain additional insight into key areas (e.g., methods used to require providers to carry out tobacco control activities, barriers plans face in addressing tobacco control); and four were added to enhance understanding of plan characteristics (e.g., accreditation status, use of Health Plan Employer Data and Information Set [HEDIS] data). Because of feedback provided during pretesting, most survey questions focused on smoking cessation despite recognition that *tobacco cessation* and *tobacco control* are more encompassing terms. The 2003 ATMC survey was originally designed to capture data about both preferred provider organizations (PPOs) and health maintenance organizations (HMOs). However, feedback received during the initial review of the survey instrument suggested that the high degree of variability in the PPO industry (risk and nonrisk bearing) made it difficult for plans to reliably respond to survey questions. As a result, the 2003 ATMC survey remained focused on the HMO product (as it had been for the previous surveys) and asked respondents to answer all questions based on their best-selling commercial HMO product.

All analyses were performed with SPSS software (SPSS, Inc, Chicago, Ill). Chi-square tests and *t* tests were used for comparisons, and results of these tests were considered statistically significant when the corresponding *P* value was .05 or less. Consistent with previous years, the data were unweighted to best describe the policies and practices of health insurance plans.

## Results

Of the 247 health plans in the sample, 32 were excluded because they were no longer in business or did not offer a commercial HMO product. Of the 215 valid plans, 160 (74%) completed and returned the survey. Collectively, the 160 plans represented more than 60 million HMO members. Respondent plans were predominantly network (48%) and mixed models (33%). Sixty-eight percent were for-profit and publicly held, 8% were for-profit and privately held, 23% were not-for-profit, and 1% were mutual companies. A comparative analysis of respondents and nonrespondents indicated that there were no significant differences in size, tax status, or predominant model type between respondents and nonrespondents.

Among respondent plans, 67% reported having a written clinical guideline for smoking cessation (Table 1). Among these plans, approximately one third reported using either the 2000 U.S. Public Health Service (PHS) Guideline on Tobacco Use and Dependence (8) or the 1996 Agency for Health Care Policy and Research (AHCPR) Practice Guideline on Tobacco Cessation (9). More than one fourth of plans reported having a guideline that had been internally developed. Among plans that reported having an internally developed guideline, almost all (92%) reported that their guideline was based on either the PHS or AHCPR guideline (data not shown). In addition, more than one third of plans with a guideline reported that their guideline came from another source (e.g., disease-management vendors, state coalitions, collaborations).

Almost all plans indicated that they could identify some or all individual plan members who smoke (Table 1). Although only 3% of plans indicated that they could identify all members who smoke, 89% reported that they could identify at least some of their members who smoke (data not shown). Among the plans that reported being able to identify smokers, the most common data sources were health-risk appraisals and telephone surveys. A relatively small percentage of plans (12%) reported using enrollment data to identify smokers. The same percentage of plans reported being able to identify smokers through registries that documented smoking status.

The vast majority (88%) of respondent plans indicated that they provided full coverage (defined as no additional charge for the member outside of the member's normal copayment for office visits) for at least one type of pharmacotherapy used for tobacco cessation (data not shown). Bupropion in the form of Wellbutrin was the most commonly covered pharmacotherapy (83%) (Table 1).

Full coverage for at least one type of behavioral intervention used for tobacco cessation was reported by most (72%) health plans (data not shown). Self-directed online resources were the most commonly covered behavioral interventions, followed by other self-help materials, individual counseling during pregnancy, and telephone counseling (Table 1).

Health plans reported having various strategies to encourage members to stop smoking during times that might be considered important teachable moments (Table 1). Almost all plans (91%) reported having a strategy for addressing smoking cessation while a member was participating in one of the plan's disease-management programs. Most health plans also reported having a strategy for addressing smoking cessation during pregnancy (69%), during treatment for chronic illnesses (65%), and following a myocardial infarction (56%).

Plans reported using several types of strategies with providers and their office staff to encourage smoking cessation among plan members. Approximately half of plans reported offering provider education, and almost one fourth of plans reported using provider prompts and reminders (Table 1). Provider prompts and reminders were coupled with provider education by 21% of plans (data not shown).

Although health plans reported various health system barriers to their ability to effectively address tobacco control, the most common barriers were related to resources (e.g., inadequate staff, funding, competing priorities) and system issues (e.g., poor data collection, reporting, record maintenance). Other important barriers reported by more than half of plans included lack of provider compliance, lack of purchaser demand, and delayed economic return on investment (Table 1).

Approximately 39% of plans reported that they currently perform some type of ROI analysis on at least some of their tobacco cessation activities (data not shown). Almost all plans (94%) indicated that they would be interested in using an ROI analysis tool designed for tobacco cessation if one were available.

Several tobacco control activities seem to be more likely to occur in larger plans than in smaller plans (Table 2). Based on the enrollment distribution of health plans in our sample, we defined larger plans as those with more than 250,000 members and smaller plans as those with 250,000 members or fewer. Larger plans were more likely than smaller plans to have a written clinical guideline for smoking cessation (*P* < .001) and to have a strategy for addressing smoking cessation during specific times, such as during adolescence, pregnancy, and postpartum visits and pediatric visits, after a myocardial infarction, and during treatment for other chronic illness (*P* ranged from < .001 to .04). We found no differences in the extent to which smaller and larger plans provided full coverage for pharmacotherapies used for smoking cessation. Although smaller plans were more likely to report providing full coverage for some types of behavioral interventions, such as telephone counseling and face-to-face counseling (*P* < .001), larger plans were more likely to report providing full coverage for self-help materials (*P* = .01) and individual counseling of pregnant women (*P* = .02). Smaller plans were also more likely to report having annual or lifetime limits on coverage for smoking cessation interventions (*P *<.001).

Although the survey instruments used in the 1997, 2000, 2002, and 2003 ATMC surveys were not identical, a core set of questions on pharmacotherapies, behavioral health, and smoking cessation strategies remained unchanged (Table 3). The percentage of plans that provided full coverage for any type of pharmacotherapy used for smoking cessation increased from 25% to 88% from 1997 to 2003 (*P* < .001). A large increase was also noted in the percentage of plans able to identify individual smokers: from 15% in 1997 to 91% in 2003 (*P* < .001).

Full coverage of behavioral interventions, such as counseling and self-help materials, fluctuated from 1997 to 2003 (Table 3). For example, more than half of the plans in the 1997 and 2000 surveys reported full coverage for self-help materials, but only one fourth of plans reported similar coverage in 2002, and the percentage of plans reporting full coverage for self-help materials increased to nearly 50% in 2003. Similarly, the 2002 survey indicated a statistically significant increase in the number of plans providing full coverage for telephone counseling, but the results of the 2003 survey showed a decrease in coverage (although the changes were not statistically significant across all 4 years of the survey).

From 1997 to 2003, the percentage of plans with strategies to address smoking cessation after a myocardial infarction increased from 22% to 56% (*P* < .001) and from 23% to 65% during treatment for other chronic diseases (*P* < .001) (Table 3). Increases in the percentage of plans with a strategy to address smoking cessation during postpartum visits (to prevent relapse) were found from 1997 to 2002 and sustained in 2003 (*P* = .03).

## Discussion

From 1997 to 2003, health plans demonstrated increasing use of evidence-based programs and clinical guidelines to address tobacco use. Clinical guidelines detail the most effective options for helping patients to quit smoking, and use of strategies recommended in clinical guidelines is associated with greater success in helping smokers to quit (9,10). Although most health plans reported having a written clinical guideline for tobacco cessation, it is possible that even more plans address tobacco cessation within other clinical guidelines used for managing or treating conditions in which tobacco use is identified as a comorbidity or risk factor (i.e., asthma, heart disease, and diabetes).

Slightly more than one fourth of the plans with a clinical guideline for tobacco cessation reported using the PHS guideline. Among plans that indicated that they use an internally developed guideline, almost all (92%) reported that their guideline was based on either the PHS or AHCPR guideline. Interestingly, nearly two thirds of plans reported that their written clinical guideline was based on or developed from a source other than the PHS or AHCPR. A review of the qualitative data provided by plans in response to this question indicated that many of the plans are using guidelines developed by disease-management vendors and various state coalitions and collaborations. This finding lends further support to the idea that health plans may be increasingly incorporating their tobacco cessation activities into the broader set of activities and guidelines that they use for the management of diseases related to tobacco use (i.e., asthma, heart disease, and diabetes).

Plans have shown tremendous improvement since 1997 in identifying individual plan members who smoke. The ability to identify smokers is an important indicator of a plan's ability to remind or prompt providers to discuss or advise patients about smoking cessation and also communicate with members about their health plan's cessation programs and benefits. Provider reminders are considered to be an effective strategy for supporting smoking cessation and are recommended by the Task Force on Community Preventive Services (10).

In the 2003 ATMC survey, the response choices to the question "Can your plan identify individual members who smoke?" were revised slightly to allow plans to indicate whether they could identify *all* members who smoke or *some* members who smoke. The results of the 2003 survey indicate that although almost all plans can identify individual members who smoke, only 3% reported being able to identify *all* members who smoke, and 89% reported being able to identify *some* members who smoke. Information provided by plans on the methods they use to identify smokers also indicates that they are most likely to identify subgroups of smokers (i.e., people who respond to health-risk appraisals or surveys). Although it would be ideal for plans to identify all smokers and intervene with each individually, identifying *all* members of any subgroup engaging in a health behavior would be a challenge for any organization.

The number of health plans providing full coverage for any type of pharmacotherapy for tobacco cessation has more than tripled since 1997. In the 2003 ATMC survey, almost nine out of 10 plans reported providing full coverage for at least one type of pharmacotherapy for tobacco cessation. Consistent with recommendations based on the effectiveness of various prescription and over-the-counter tobacco cessation first-line pharmacotherapies (9), most plans reported providing full coverage for at least one form of bupropion (Wellbutrin). The significant increase in the number of plans that provide full coverage for at least one type of pharmacotherapy for tobacco cessation is well aligned with the growing body of literature indicating that reduced out-of-pocket cost is associated with greater use of tobacco cessation programs and services (11,12) and may lead to increased rates of cessation (13).

Given the literature citing the effectiveness of telephone counseling and indicating that smokers are more likely to use telephone counseling than to participate in individual or group counseling sessions (14,15), it is not surprising that more plans in 2002 reported offering full coverage for telephone counseling than in 1997. However, the results of the 2003 ATMC survey indicate that fewer plans in 2003 are providing full coverage for telephone counseling than in 2002. Although the survey did not assess reasons for offering or not offering specific types of interventions, it is possible that increased availability of local or state-sponsored quit lines has resulted in less need for health plans to provide coverage for telephone counseling.

The fact that 91% of plans reported having a strategy for addressing tobacco cessation with patients already participating in disease-management programs underscores the importance of promoting disease-management programs as a vehicle for addressing tobacco cessation. In environments where numerous health improvement programs must compete for limited resources, the ability to effectively address tobacco cessation within the context of other programs may be strategically and clinically important. Indeed, most plans have strategies for addressing tobacco cessation during pregnancy, when a patient is being treated for a chronic illness, and after acute events such as myocardial infarction, suggesting that plans are moving in this direction.

Health plans continue to report that resource limitations, including too few staff and inadequate funding, are leading barriers to adequately addressing tobacco control. Although just over one third of plans reported conducting any type of ROI analysis on their tobacco cessation activities in 2003, there was widespread interest in identifying and using ROI analysis tools for tobacco cessation. Fortunately, research supported by the NTAO has recently resulted in the development of a Web-based ROI analysis tool for smoking cessation interventions based primarily on smoking-attributable costs to health plans (16). The ROI tool should be especially useful to health plans that need to rationalize their investments in smoking cessation interventions or convince purchasers of the value of such interventions.

### Limitations

The ATMC survey and its findings have limitations. The response rate of approximately 74% is respectable but still leaves open the possibility of selection bias. Although no significant differences were detected between respondents and nonrespondents on three key characteristics (size, tax status, and predominant model type), respondents may possibly differ from nonrespondents in ways that were not measured (e.g., level of interest, commitment to tobacco control). Another limitation to the ATMC survey is that the psychometric properties of the questionnaire were not tested to assess reliability or validity. However, the survey design process did include pretesting to increase the probability of including questions that were reliable and likely to yield valid responses. Additionally, we identified a potential limitation of the 1997 survey — it did not include a frame of reference for product type — and corrected all subsequent ATMC surveys accordingly. Based on inquiries made to plans following the 1997 survey, we learned that when a frame of reference is not provided, the tendency is to base answers on the best-selling HMO product, and this is what respondents were explicitly asked to do in 2000, 2002, and 2003. However, there is still a possibility that the change in frame of reference contributed to some of the differences in survey findings between the 1997 survey and more recent surveys (but not between the 2000, 2002, or 2003 surveys).

Few surveys other than the ATMC surveys have been designed to assess tobacco control practices and policies of health plans. Of those that have been conducted and published, some have focused on plans that operated only in a single state (12,17); some have included only a narrow subset of plans (i.e., well-established, nonprofit plans with a history of offering tobacco cessation programs) (18); and others have collected information only about subsets of smokers within a plan (i.e., pregnant women) (19,20). Despite their more limited scope, these surveys have yielded data comparable to the data from the ATMC surveys.

### Conclusion

The results of the 2003 ATMC survey indicate that an increasing number of health plans are using evidence-based approaches and strategies to address tobacco use among members. Although almost all plans reported that their tobacco control activities were limited by resource and systems barriers, they have been able to sustain the improvements made since 1997. Even so, many plans may benefit from taking advantage of the recently developed ROI analysis tool to leverage the body of literature that supports the cost-effectiveness of tobacco cessation treatment and advocate for the resources necessary to sustain their tobacco control activities (8,21-23).

As others have previously noted, health plans play an important role in tobacco control (24). In particular, plans continue to be in a unique position to implement operational policies and programs that can reduce the prevalence of tobacco use and improve the health of millions of people. In addition to their role in sustaining and expanding access to tobacco cessation treatments and services, health plans should continue to model new tobacco cessation benefits, promote them widely to their membership, and influence large purchasers of health care services by communicating the value of tobacco cessation services. New opportunities to participate in policy initiatives that support tobacco control and promote public health are essential next steps to maintain the availability of these services over the long term.

It is unclear whether the findings from the 2003 ATMC survey apply to other forms of health insurance, such as PPOs. Unlike HMOs, which have traditionally emphasized preventive health care and wellness activities such as smoking cessation, PPOs have emphasized network size, expertise, and discounted access to network providers for members. Although some health insurance companies are likely to offer the same tobacco control programs in both their PPO and HMO products, others may vary their tobacco control programs and policies by product or purchaser.

Nevertheless, the lessons learned in tobacco control should be applied to other areas where behavioral health modification is a core component in the treatment of the illness or condition. We agree with others who have stated that one of the most important lessons to be learned from tobacco control is that tackling similar conditions (e.g., obesity) will require a sustained, thoughtful, well-resourced, multidimensional effort (25). Additional lessons may include recognition of the importance of being able to identify individuals in need of services, offering coverage for effective pharmacotherapies and treatments, and incorporating programs (such as those for tobacco cessation and obesity) into existing disease-management programs for which tobacco use and obesity are risk factors or common comorbidities.

The period from 1997 to 2003 was an active and significant time for tobacco control at the local, state, and national levels. During these 7 years, health plans accomplished a great deal and demonstrated a strong commitment to smoking cessation with proven results. Yet there are still many important opportunities for health plans to advance their tobacco control activities and to transfer the lessons learned in tobacco control to other important public health priorities. Health plans and other stakeholders should look ahead to the coming years for opportunities to continue their collaborative efforts to improve the health of individuals and populations.

## Figures and Tables

**Table 1:** Results from the 2003 Addressing Tobacco in Managed Care Survey (N = 160)

**Activity**	**% Yes**
**Plan has a written clinical guideline for smoking cessation**	66.6
**Among plans with a written clinical guideline for smoking cessation**	
Plan uses the 2000 U.S. Public Health Service Guideline (8)	28.3
Plan uses an internally developed clinical guideline	27.4
Plan uses the 1996 Agency for Health Care Policy and Research Guideline (9)	7.5
Plan uses a guideline from some other source	36.8
**Plan is able to identify some or all members who smoke**	91.1
**Among plans that can identify smokers, data sources used by plans to identify individual members who smoke**	
Health-risk appraisal	64.7
Telephone survey	59.3
Mail-based survey	34.1
Medical record review (random sample)	18.7
Administrative data review	16.5
Electronic medical record	15.4
Enrollment information	12.1
Registry containing smoking status	12.1
**Plan provides full coverage for**	
Bupropion (as Wellbutrin)	83.3
Bupropion (as Zyban)	29.5
Prescription NRT nasal spray	19.2
Prescription NRT inhaler	19.2
Prescription NRT patches	18.6
Over-the-counter NRT patches	9.6
Over-the-counter NRT gum	7.7
Over-the-counter NRT lozenges	6.4
**Plan provides full coverage for**	
Self-directed, online resources (interactive and noninteractive)	56.4
Self-help materials (booklets, videos, audiotapes, customized mailings)	45.5
Individual counseling of pregnant women	44.2
Telephone counseling	42.3
Individual face-to-face counseling	35.9
Group counseling or classes	21.2
**Plan has annual or lifetime limits on coverage for smoking cessation interventions**	19.3
**Plan allows patients to self-refer to smoking cessation services**	48.8
**Plan has a strategy to address smoking cessation**	
During participation in disease-management programs	91.0
During pregnancy	68.6
During treatment for other chronic illness	64.7
After myocardial infarction	56.4
During postpartum visits (relapse prevention)	46.8
During adolescence	32.1
During pediatric visits (secondhand smoke)	29.5
During hospitalizations	11.5
**Plan funds a full- or part-time tobacco control program staff position**	16.1
**Plan used the following strategies with members in the past year to inform them about cessation benefits or encourage them to take advantage of covered treatments**	
General member education (e.g., newsletters, Web site, announcements)	60.0
Customized member education (e.g., mailings directed at members meeting criteria or conditions)	31.0
Increased availability of smoking cessation programs and interventions	22.6
Discounts or reimbursements for NRT	21.3
Discounts or reimbursements for community resources	14.8
**Plan used the following strategies with providers, office staff, or both in the past year to promote smoking cessation**	
Provider education	51.9
Prompts and reminders to encourage providers to address tobacco control	22.4
Elimination of preauthorization requirements for smoking cessation interventions	9.1
Incentives for providers and their staff to effectively address tobacco	7.7
Increased reimbursement for smoking cessation counseling and assistance	3.9
Increased amount of time that providers spend with patients	0.6
**Barriers to addressing tobacco control among plans**	
Resource barriers (e.g., staff, funding, competing priorities)	92.9
System barriers (e.g., data collection, data reporting, record maintenance)	87.7
Delayed economic return on investment	61.3
Lack of purchaser demand	54.5
Lack of provider compliance	54.2
Lack of patient demand	42.6

NRT indicates nicotine replacement therapy.

**Table 2:** Tobacco Control Activities by Size of Health Plan, 2003 Addressing Tobacco in Managed Care Survey (N = 160)

**Activity**	**≤250,000 Plan Members (N = 63), % Yes**	**>250,000 Plan Members (N = 97), % Yes**	***P* Value^a^**
**Plan has a written clinical guideline for smoking cessation**	25.4	61.5	<.001
**Plan provides full coverage for**			
NRT over-the-counter gum	5.1	9.3	.34
NRT over-the-counter patches	8.5	10.3	.71
NRT inhalers	20.3	18.6	.78
NRT nasal spray	20.3	18.6	.78
Bupropion (as Zyban)	37.3	24.7	.10
Bupropion (as Wellbutrin)	83.1	83.5	.94
**Plan provides full coverage for**			
Telephone counseling	74.6	22.7	<.001
Face-to-face counseling	69.5	15.5	<.001
Group counseling or classes	28.8	16.5	.07
Individual counseling of pregnant women	32.2	51.5	.02
Self-help materials	32.2	53.6	.01
**Plan has annual or lifetime limits on coverage for smoking cessation interventions**	25.8	15.5	<.001
**Plan allows patients to self-refer to smoking cessation services**	50.9	47.0	.67
**Plan is able to identify individual members who smoke**	88.4	92.8	.56
**Plan has a strategy to address smoking cessation**			
During adolescence	22.0	38.1	.04
During pregnancy	40.7	85.6	<.001
During postpartum visits (relapse prevention)	11.9	68.0	<.001
During pediatric visits (second hand smoke)	13.6	39.2	.001
After myocardial infarction	25.4	75.3	<.001
During treatment for other chronic illness	37.3	81.4	<.001
During hospitalizations	15.3	9.3	.26
**Plan funds a tobacco control program staff position**	22.4	12.4	.14

NRT indicates nicotine replacement therapy.

a Proportions were compared using the chi-square test; results were considered statistically significant at *P* ≤.05.

**Table 3:** Comparison of Data from the 1997, 2000, 2002, and 2003 Addressing Tobacco in Managed Care Surveys^a^

**Activity**	**1997 Respondents (N = 323), % Yes**	**2000 Respondents (N = 85), % Yes**	**2002 Respondents (N = 152), % Yes**	**2003 Respondents (N = 160), % Yes**	***P* Value^b^**
**Plan provides full coverage for**					
Any pharmacotherapy for smoking cessation	25.0	20.0	88.8	87.8	<.001
Bupropion (as Zyban)	17.6	37.2	41.1	29.5	.10
Any over-the-counter NRT	6.6	14.9	8.6	9.6	<.001
NRT only with program enrollment	25.0	26.0	10.8	19.3	.01
**Plan provides full coverage for**					
Telephone counseling	32.8	36.8	51.7	42.3	.07
Face-to-face counseling	26.6	23.6	41.1	35.9	.04
Group counseling or classes	35.7	37.0	15.9	21.2	.002
Self-help materials	54.1	56.6	25.8	45.5	<.001
Any behavioral therapy or pharmacotherapy	75.0	94.4	98.0	96.2	<.001
**Plan is able to identify individual members (some or all) who smoke**	14.9	27.1	71.7	91.1	<.001
**Plan has a specific strategy to address smoking cessation**					
During adolescence	17.6	24.2	28.9	32.1	.46
During pregnancy	45.0	59.0	56.6	68.6	.08
During postpartum visits	13.6	30.5	46.7	46.8	.03
During pediatric visits	15.8	17.3	28.3	29.5	.10
After myocardial infarction	21.7	27.2	46.7	56.4	<.001
During treatment for chronic illness	22.6	31.3	52.0	64.7	<.001
**Plan funds a full- or part-time tobacco control program staff position**	7.7	23.5	19.1	16.1	<.001

NRT indicates nicotine replacement therapy.

a Although the survey instruments used in the 1997, 2000, 2002, and 2003 Addressing Tobacco in Managed Care surveys were not identical, a core set of questions on pharmacotherapies, behavioral health, and smoking cessation strategies remained unchanged.

b Proportions were compared for 1997 and 2003 surveys using the chi-square test; results were considered statistically significant at *P* ≤.05.
